# Neuropilin-1 drives tumor-specific uptake of chlorotoxin

**DOI:** 10.1186/s12964-019-0368-9

**Published:** 2019-06-17

**Authors:** Sharon McGonigle, Utpal Majumder, Donna Kolber-Simonds, Jiayi Wu, Andrew Hart, Thomas Noland, Karen TenDyke, Daniel Custar, Danyang Li, Hong Du, Maarten H. D. Postema, W. George Lai, Natalie C. Twine, Mary Woodall-Jappe, Kenichi Nomoto

**Affiliations:** 1 Oncology Business Group, Eisai Inc, 4 Corporate Drive, Andover, MA 01810 USA; 2 Present address: Solid Biosciences, 141 Portland Avenue, Cambridge, MA 02139 USA; 3 Present address: Wave Life Sciences, 115 Hartwell Ave, Lexington, MA 02421 USA; 4 Present address: Mersana Therapeutics, 840 Memorial Drive, Cambridge, MA 02139 USA; 5 Eisai Inc, 100 Tice Blvd, Woodcliff Lake, NJ 07677 USA

**Keywords:** Chlorotoxin, Neuropilin-1, Tumor, Uptake, Peptide-drug-conjugate

## Abstract

**Background:**

Chlorotoxin (Cltx) isolated from scorpion venom is an established tumor targeting and antiangiogenic peptide. Radiolabeled Cltx therapeutic (^131^I-TM601) yielded promising results in human glioma clinical studies, and the imaging agent tozuleristide, is under investigation in CNS cancer studies. Several binding targets have previously been proposed for Cltx but none effectively explain its pleiotropic effects; its true target remains ambiguous and is the focus of this study.

**Methods:**

A peptide-drug conjugate (ER-472) composed of Cltx linked to cryptophycin as warhead was developed as a tool to probe the molecular target and mechanism of action of Cltx, using multiple xenograft models.

**Results:**

Neuropilin-1 (NRP1), an endocytic receptor on tumor and endothelial cells, was identified as a novel Cltx target, and NRP1 binding by Cltx increased drug uptake into tumor. Metabolism of Cltx to peptide bearing free C-terminal arginine, a prerequisite for NRP1 binding, took place in the tumor microenvironment, while native scorpion Cltx with amidated C-terminal arginine did not bind NRP1, and instead acts as a cryptic peptide. Antitumor activity of ER-472 in xenografts correlated to tumor NRP1 expression. Potency was significantly reduced by treatment with NRP1 blocking antibodies or knockout in tumor cells, confirming a role for NRP1-binding in ER-472 activity. Higher cryptophycin metabolite levels were measured in NRP1-expressing tumors, evidence of NRP1-mediated enhanced drug uptake and presumably responsible for the superior antitumor efficacy.

**Conclusions:**

NRP1 was identified as a novel Cltx target which enhances tumor drug uptake. This finding should facilitate tumor selection for chlorotoxin-based therapeutics and diagnostics.

**Electronic supplementary material:**

The online version of this article (10.1186/s12964-019-0368-9) contains supplementary material, which is available to authorized users.

## Background

Chlorotoxin (Cltx) is a small basic peptide first isolated from the venom of the scorpion *Leiurus quinquestriatus* and named for its ability to block chloride channels and cause neurotoxicity [1]. Injection of crayfish or cockroaches with purified peptide results in paralysis of these arthropods, presumably through blocking chloride channels in their muscles [1]. The tumor targeting property of Cltx was first described in 1998 by Soroceanu et al. [2]. They reported high affinity binding of Cltx to glioma cells with minimal binding to normal brain cells, as well as targeting of glioma tumors in xenografted mice using radiolabeled Cltx. Their findings ultimately led to the development of radiolabeled Cltx (^131^I-TM601, TM601 refers to chemically synthesized Cltx) as a clinical candidate for glioblastoma [3]. Promising data was generated in early glioma trials; ^131^I-TM601 also demonstrated tumor-specific uptake beyond glioma in multiple diverse cancer indications in preliminary clinical investigations [3, 4]. Additionally, Cltx that has been covalently linked to indocyanine green, a near-infrared fluorophore (tozuleristide, BLZ-100), is currently in clinical development for intraoperative visualization of human solid tumors ([5], https://clinicaltrials.gov/).

Tumor-specific binding by Cltx, first described in glioma, was more broadly observed in a variety of diverse tumor types, in contrast to normal tissue which consistently remained refractory to Cltx binding [6]. Cltx’s tumor selectivity was originally attributed to binding of chloride ion channels (CLC-3) in glioma cells, and functionally, Cltx inhibited migration and invasion by glioma cells in a dose dependent manner [7]. Subsequently, MMP2, in complex with MMP14, TIMP and α_v_β_3_ was identified as a receptor for Cltx and proposed to play a role in the observed anti-invasive activity [8]. Treatment of cells with Cltx resulted in internalization and down regulation of cell surface levels of both putative targets, MMP2 and CLC-3 [9]. Nonetheless, in follow up studies a direct interaction between Cltx and MMP2 could not be established [10].

Further investigation revealed that Cltx will bind and undergo internalization by proliferating endothelial cells, the first reported interaction of the peptide with a normal, untransformed cell type. These findings led to the identification of annexinA2 as an additional target of Cltx; annexinA2 is expressed on the surface of many tumor cells as well as vascular endothelial cells [11, 12]. The ability of Cltx to inhibit angiogenesis, presumably as a consequence of binding to endothelial cells, was demonstrated in various animal models [12, 13]. Together, these data advanced the possibility of using Cltx therapeutically to target tumor cells through delivery of conjugated cytotoxic drugs, in addition to targeting angiogenesis as a non-conjugated peptide. Individually, none of the several targets proposed thus far provided a reasonable explanation for the extensive pleiotropic effects documented for Cltx.

A more complete understanding of its mechanism of action is essential to fully realize the broad potential of Cltx as a therapeutic, a diagnostic, or a tumor targeting drug delivery vehicle. Toward this objective, we describe the novel peptide drug conjugate (PDC) ER-472, comprised of Cltx linked to a novel cryptophycin analog as cytotoxic warhead. ER-472 was used as a tool to comprehensively investigate the true molecular target and mechanism of action of Cltx. In studies described herein the endocytic receptor neuropilin-1 (NRP1) was identified as a novel Cltx binding partner. A C-terminal arginine residue proved essential for binding of peptides to NRP1. Native Cltx with amidated arginine at its C-terminus did not bind NRP1 but acted as a cryptic peptide; its metabolism to peptides bearing a free C-terminal arginine and the capacity to bind NRP1 took place in the tumor microenvironment. Cltx-NRP1 binding enhanced tumor uptake of ER-472 active metabolite that was blocked by anti-NRP1 antibody treatment or NRP1 knockdown and resulted in diminished ER-472 antitumor activity. This newly identified targeting mechanism of Cltx via NRP1 binding appeared to be separate and distinct from the neurotoxic effects of Cltx and will be useful in selection of tumors for Cltx application.

## Methods

### Reagents

Native Cltx (Cltx-CONH_2_) was synthesized at CPC Scientific (Sunnyvale, CA) by routine methods and stored dry at -20 °C. Recombinant Cltx expressed in *E.coli* (Cat# RTC-450 Alomone Labs, Jerusalem, Israel) has a carboxylated C-terminal arginine residue (Cltx-COOH). Cltx peptide was dissolved in PBS for routine studies. The PDC ER-472 and compounds ER-271 and S-methyl cryptophycin were synthesized at Eisai; details provided in [14]. For in vivo administration all compounds were formulated in 10% ethanol, 5% Tween 80 and 85% saline. For in vitro studies PDC was dissolved in PBS.

BxPC-3 cells (BW125058) were from Perkin Elmer (Waltham, MA), MIA PaCa-2 (ATCC Cat# CRM-CRL-1420, RRID:CVCL_0428) and PC-3 (ATCC Cat# CRL-7934, RRID:CVCL_0035) were from ATCC. All cell lines were maintained according to their instructions. Cells were used within a short time of receipt, or cell line authenticity was verified by STR typing (IDEXX BioResearch, Columbia, MO) and all cells were certified mycoplasma-free prior to culture (qPCR at Charles River Labs, Wilmington, MA).

### In vivo studies

All studies were performed according to IACUC approved protocols. The general health of mice was monitored daily. Tumor volume was determined by caliper measurements (mm), using the formula (l x w^2^)/2 = mm^3^, where l and w refer to the larger and smaller perpendicular dimensions collected at each measurement. Tumor dimensions were recorded twice per week starting when tumors reached an approximate size of 100 to 150 mm^3^. For xenograft models MIA PaCa-2, BxPC-3 and PC-3, cells were injected subcutaneously near the right axillary area, into female Nu/Nu mice (Charles River Labs), approximately 6–8 weeks old. CRISPR engineering of the PC-3 cells resulted in isogenic cell lines that were less tumorigenic than parental cells and therefore these xenograft models were performed in NOD SCID mice. For efficacy studies treatments were initiated when the average tumor size was approximately 200 mm^3^. ER-472 was administered at ¼, ½ or full MTD dose (0.6, 1.2 or 2.3–2.5 mg/kg respectively) once every 4 days, 3 times (Q4Dx3).

For studies to detect tumor levels of active metabolite, a single dose of drug was administered when tumors volumes reached ~ 300 to 500 mm^3^; animals were euthanized and tumors harvested at multiple time points from 5 min to 96 h post-dose (*n* = 3 mice per group). Tumors were cryofractured using the Cryoprep System (Covaris, Inc.), lysed 3:1 (v:w) in PBS containing 1x Halt™ Protease Inhibitor Cocktail (Thermo Scientific, Cat# 78430), and were extracted 3:1 with methanol:acetonitrile 1:1 (v:v) containing 0.1% acetic acid. ER-472 and S-methyl metabolite were then quantified using liquid chromatography coupled with tandem mass spectrometry analysis (LC-MS/MS).

To detect Cltx and derived peptides in PC-3 tumor lysate, Cltx peptide was administered at 150 mg/kg and tumors were harvested 1 h later. Following cryofracture, tumors were lysed in Super B buffer (Covaris, Cat# 520112) containing protease inhibitors, centrifuged, dried and re-constituted in acetonitrile, ammonium bicarbonate, pH 8. Proteins and peptides were reduced with dithiothreitol and the cysteine residues were capped with iodoacetamide prior to LC-MS/MS analysis. BiopharmaLynx™ version 1.33 software (Waters) was used to analyze and identify Cltx-related peptides.

### In vitro studies

Cell proliferation assays were performed in 96 well plates with 3000 cells seeded per well; ER-472 was added at various concentrations and plates were incubated for various time points. Cell growth was assessed using the CellTiter-Glo® luminescent cell viability assay (Promega, Cat# G7573).

### Ex vivo studies

Tumors were harvested from various models and flash frozen prior to lysis and analysis of thiol, protein or mRNA levels. For thiol and western studies, total protein was measured in tumor lysates following cryofracture and resuspension in lysis buffer, using the Pierce™ BCA protein assay kit (Thermo Fisher Scientific Cat# 23225). Antibodies used in westerns were NRP1 (Cell Signaling Technology Cat# 3725, RRID:AB_2155231), CLC-3 (D8Y5Q) (Cell Signaling Technology, Cat# 13359), MMP2 (D204T) (Cell Signaling Technology, Cat# 87809), AnnexinA2 (D11G2) (Cell Signaling Technology, Cat# 8235) and tubulin (Abcam Cat# ab6160, RRID:AB_305328). In certain blots recombinant human proteins were included as positive controls: NRP1, Phe22-Lys644 (Cat# 3870-N1, R&D Systems), CLC-3 (Cat# H00001182-Q, Novus Biologicals), MMP2 (Cat# 902-MP, R&D Systems) and AnnexinA2 (Cat# 9409-AN, R&D Systems. Immune complexes were visualized with appropriate IRDye® 680RD and 800CW-conjugated secondary antibodies (LI-COR Biosciences) using an Odyssey Infrared Imaging System (LI-COR Biosciences). RNA was extracted from excised tumors (*n* = 4) using the RNeasy Mini Kit (Qiagen, Germany). Total RNA (2 μg) was converted to cDNA using SuperScript VILO Master Mix (Life Technologies, USA) and amplified using Applied Biosystems TaqMan expression assays using RNA-specific primers for NRP1 (Hs00826128_m1), GUSB (Hs99999908_m1), and HPRT1 (Hs00000009_m1). QPCR was performed with TaqMan Fast Advanced Master Mix (Life Technologies, USA) on the Applied Biosystems 7900 instrument and relative gene expression was calculated after normalization against reference genes GUSB and HPRT1 using the using the 2 ^− ΔΔCT^ method in the Applied Biosystems SDS 2.4 software.

### Peptides for NRP1 binding studies

Octet binding studies required biotinylation of Cltx and derived peptides for loading onto streptavidin biosensors. Trypsin, (Promega Cat# V5280) (~ 40 μg/mL) was added to Cltx biotinylated at K27 or N-terminus (10 mg/mL) and incubated for 24 h at 37 °C, pH 8.5. The resulting peptides were separated by preparative HPLC and peptide fragments were characterized by MALDI. Small linear Cltx-derived peptides were also synthesized by routine methods and were biotinylated at the N-terminus. In some cases, peptides were reduced with TCEP at pH 8 and the cysteine residues were capped using iodoacetamide.

### Bio-layer interferometry

Assessment of binding between NRP1and Cltx was performed by Bio-Layer Interferometry (BLI) using the Octet Red96 System (ForteBio, Pall). Cltx and derived fragments were captured on streptavidin biosensors (Cat# 18–5020) under approximately equivalent loading conditions; sensors were then quenched with EZ-Link Biocytin and washed in assay buffer (PBS, 1% BSA, 0.05% Tween20). Binding to NRP1 (Cat# 10011-H02H, Sino Biological Inc.) at concentrations from 0 to 1750 nmol/L was determined by association then dissociation (1 min each) in assay buffer. Binding constant (K_D_) was determined by measuring binding over the range of NRP1 concentrations (Octet Data Analysis Software version 8.2). For competition studies, binding of biotinylated VEGF165 (Cat# VE5-H8210, ACRO Biosystems) to NRP1 (390 nmol/L) was measured in the presence of Cltx-CONH_2_ or Cltx-COOH at concentrations from 50 to 800 μmol/L.

### Crayfish bioassay

The crayfish assay to determine bioactivity of Cltx was performed at ABC Labs (Columbia, MO, now EAG Labs) using an SOP generated by TransMolecular Inc. Cltx peptide (20 μg) in sterile water was injected through the ventral surface of the thorax in the region of the sub-esophageal ganglion of crayfish which were then subjected to physical challenge defined as multiple, continuous, gentle prods in random parts of the body from head to tail. The animal’s response is based on avoidance, ability to right itself, and clasping with chelae in a defensive manner. Time from injection to total incapacitation was recorded; total incapacitation is defined as the time when the tested crayfish is not responsive when physically challenged.

## Results

### Peptide drug conjugate ER-472

**Fig. 1:**
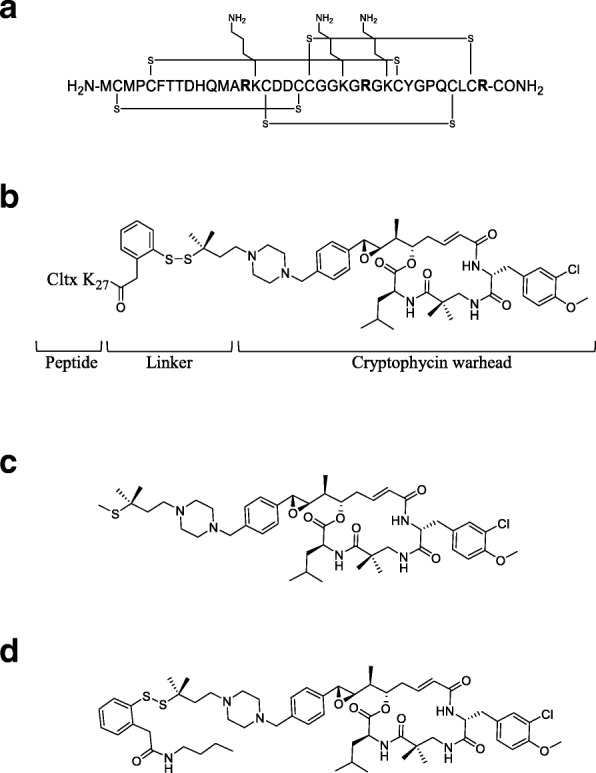
Structure of Cltx, ER-472 and related compounds. **a**, amino acid sequence of native Cltx which features 4 disulfide bonds, 3 lysine (K) and 3 arginine (R, in bold) residues, including amidated R at the C-terminus. **b**, structure of ER-472 PDC comprised of Cltx peptide linked via K27 to a novel cryptophycin analog (cytotoxic warhead), via a cleavable dimethyl disulfide linker. **c**, S-methyl cryptophycin, the active metabolite of ER-472 generated in tumor. **d**, ER-271 mimics ER-472 that lacks Cltx; composed of cryptophycin analog plus the dimethyl disulfide linker which terminates in an aryl group

The 36 amino acid sequence of Cltx is shown in Fig. 1a. Its sequence contains 2 internal arginine residues and a C-terminal arginine, which is amidated in native Cltx (Fig. 1a). To generate the peptide drug conjugate (PDC) ER-472, Cltx was conjugated at lysine 27 to a novel analog of cryptophycin via a cleavable dimethyl disulfide linker (Fig. 1b, Additional file 1: Figure S1). The maximum tolerated dose (MTD) for ER-472 was determined as 2.3 to 2.5 mg/kg following intravenous administration of conjugate every 4 days 3 times (data not shown). In vivo metabolism of ER-472 and release of the warhead cryptophycin from the PDC previously revealed that S-methyl cryptophycin is the active metabolite generated from conjugate in vivo [14], (Fig. 1c, Additional file 1: Figure S2).

### Differential antitumor activity of ER-472

**Fig. 2:**
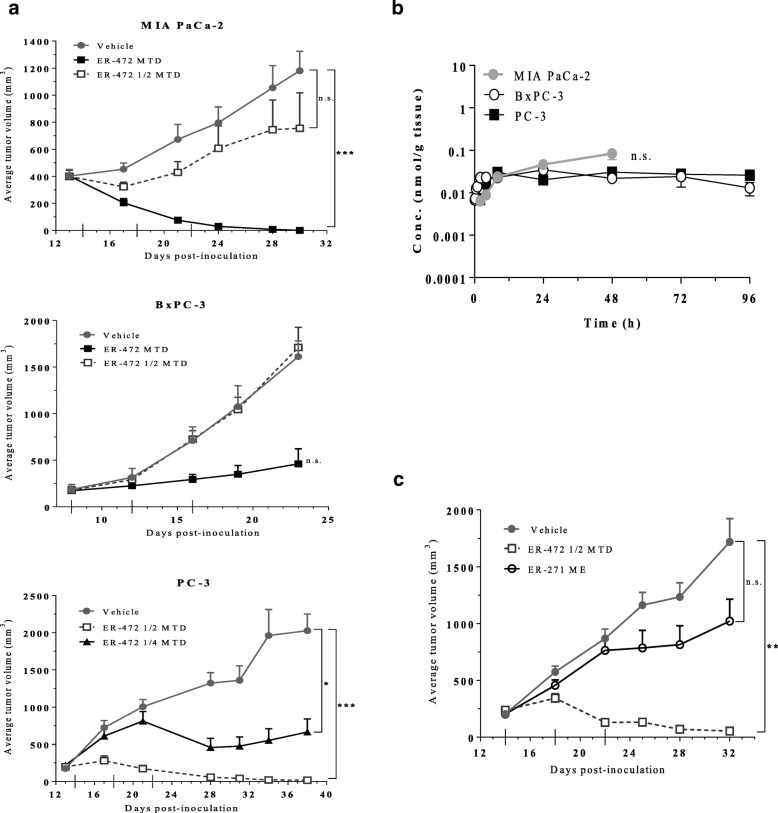
ER-472 antitumor activity and levels of active metabolite in 3 xenograft models; MIA PaCa-2, BxPC-3 and PC-3. Subcutaneous tumors were established in nude mice; treatment groups were *n* = 6 for MIA PaCa-2 and BxPC-3 and *n* = 5 for PC-3 efficacy studies and *n* = 3 for all metabolite studies. **a**, ER-472 was administered i.v. Q4Dx3 (dosing days denoted with long ticks on x-axis). Tumor volume data represents the mean ± SEM. **b**, mean active metabolite (S-methyl cryptophycin) tumor concentration versus time following a single dose of ER-472 at 2.5 mg/kg (MTD). Data represents the mean ± StdDev. **c**, antitumor activity of ER-472 versus ER-271 at molar equivalent (ME) dose in PC-3 model. *P ≤ 0.05, **P ≤ 0.01, ***P ≤ 0.001, one-way ANOVA followed by Tukey’s multiple comparison test; n.s. = not significant

Initial assessment of the in vivo antitumor effects of ER-472 in 3 xenograft models (MIA PaCa-2, BxPC-3 and PC-3) revealed differential sensitivity. At the MTD dose, ER-472 treatment resulted in significant antitumor activity in all 3 models, but at ½ MTD no antitumor activity was observed in MIA PaCa-2 or BxPC-3 xenografts (Fig. 2a). In contrast, a much wider therapeutic window was observed for ER-472 in PC-3 xenografts: tumor regression and several tumor cures were observed at ½ MTD and significant antitumor activity was also recorded at the ¼ MTD dose (Fig. 2a). The differential antitumor activity did not correlate to an inherent difference in in vitro tumor cell sensitivity to ER-472, as all 3 cell lines had comparable IC_50_ values and kinetics of cell growth inhibition (Additional file 1: Table S1). Thiol levels were also identical in the 3 tumor lysates, ruling out the possibility of differential release of active warhead from ER-472 (Additional file 1: Table S2). In addition, no correlation was observed to expression levels of any of the previously identified targets of Cltx; CLC-3, MMP2 and annexinA2 (Additional file 1: Figure S3). Unexpectedly, the generation of active metabolite S-methyl cryptophycin in tumor lysate following a single ER-472 treatment was indistinguishable in the 3 distinct models (Fig. 2b). In all 3 cases the level of active metabolite increased in the first few hours post-treatment and then remained at steady state until the last measurement (> 48 h post-dose). Active metabolite was measured following lysis of the entire excised tumor, prompting speculation that the differential antitumor activity observed might be due to differential ER-472 localization or uptake within specific cells of the tumor.

To determine if Cltx within the ER-472 conjugate was important for the wide therapeutic window observed in PC-3 xenografts, ER-271 was synthesized for comparison (Fig. 1d). ER-271 lacked Cltx but was otherwise identical to ER-472; composed of the same cryptophycin analog warhead and dimethyl disulfide linker, which in the case of ER-271 terminated in an aryl group (Fig. 1d). When PC-3 tumored mice were treated at molar equivalent doses of ER-472 (½ MTD) and ER-271, tumor regression was observed for ER-472 while only a modest effect on tumor growth was observed for ER-271 (Fig. 2c). These results suggested that the presence of Cltx within the PDC significantly enhanced its antitumor activity.

### Cltx derived peptides bind to NRP1

**Fig. 3:**
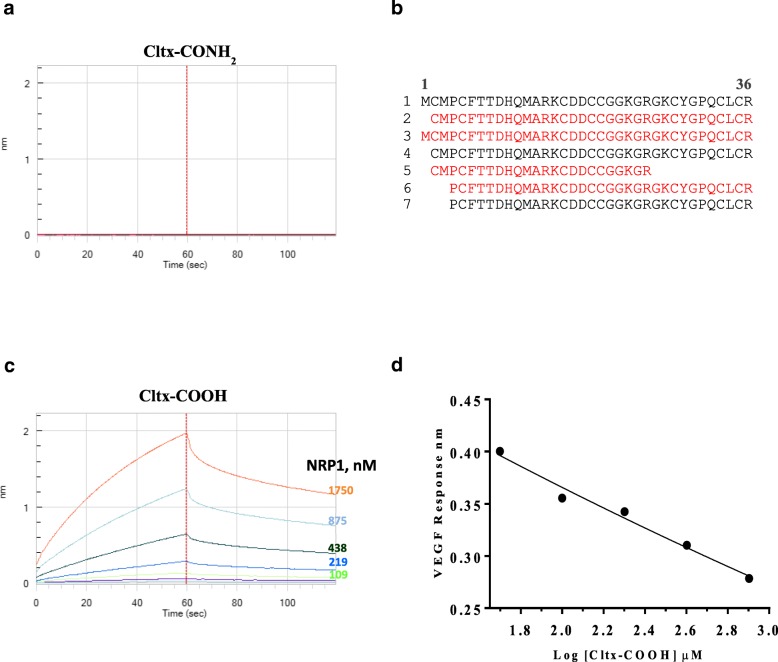
Cltx binds to NRP1 only when its C-terminal arginine is de-amidated; Cltx de-amidation occurs in tumor. Cltx-CONH_2_ (native Cltx with amidated C-terminal arginine) does not bind to NRP1 at concentrations from 0 to 1750 nM (**a**) while Cltx-COOH (Cltx with a carboxylated C-terminal arginine) demonstrated dose responsive NRP1 binding with an affinity (K_D_) of ~ 240 nM (**c**). Graphs are output from BLI assay with K_D_ determined by octet data analysis software version 8.2; graphs are representative of multiple assays. **b**, peptides identified in PC-3 tumor lysate by MS analysis 1 h post dose of Cltx, in order of abundance. Peptides represented in black or red have amidated versus de-amidated C-terminal arginine residue respectively. Peptides 1 and 3 are full length Cltx with amidated (native) versus de-amidated C-terminal arginine respectively. **d**, VEGF binding to NRP1 (fixed concentration at 390 nM) was dose dependently inhibited in the presence of increasing concentration of Cltx-COOH (0 to 800 μM), R^2^ = 0.98; suggests that Cltx binding to NRP1 occurs at VEGF binding site

NRP1 is an endocytic receptor reported to increase cellular uptake of drugs, proteins, viruses, etc. in response to ligand binding at its VEGF binding site [15–18]. Bio-layer interferometry (BLI) using the Octet system was used to examine the interaction between Cltx and NRP1. Full length native Cltx in which the C-terminal arginine is amidated (Cltx-CONH_2_) did not bind to NRP1 (Fig. 3a). To determine whether NRP1 binding peptides could be generated from Cltx, in vitro trypsinization was performed. Several Cltx-derived peptides (including tri-peptides held together by disulfide bonds) demonstrated NRP1 binding; all of these possessed at least one carboxylated C-terminal arginine residue (Table 1). Multiple additional Cltx-derived peptides generated either from enzymatic digestion of Cltx or by chemical synthesis were assessed in NRP1 binding studies (Table 1). The ability of a peptide to bind NRP1 was dictated by the absolute requirement for carboxylated arginine (R-COOH) as the C-terminal residue, with no binding observed for peptides that terminated with amidated arginine (R-CONH_2_), lysine, leucine or glutamic acid. The binding affinity of NRP1 for Cltx-derived peptides ranged from 100 to 400 nM as compared to ~ 50 nM for the native substrate VEGF (Table 1). While the C-terminal arginine appeared essential for binding to NRP1, additional peptide characteristics such as identity of preceding amino acids, peptide length, disulfide bonds and tertiary structure, etc., did not significantly impact binding affinity (Table 1). In conclusion, binding to NRP1 was observed for peptides derived from chlorotoxin that terminated with carboxylated arginine, but not for native chlorotoxin.

**Table 1:**
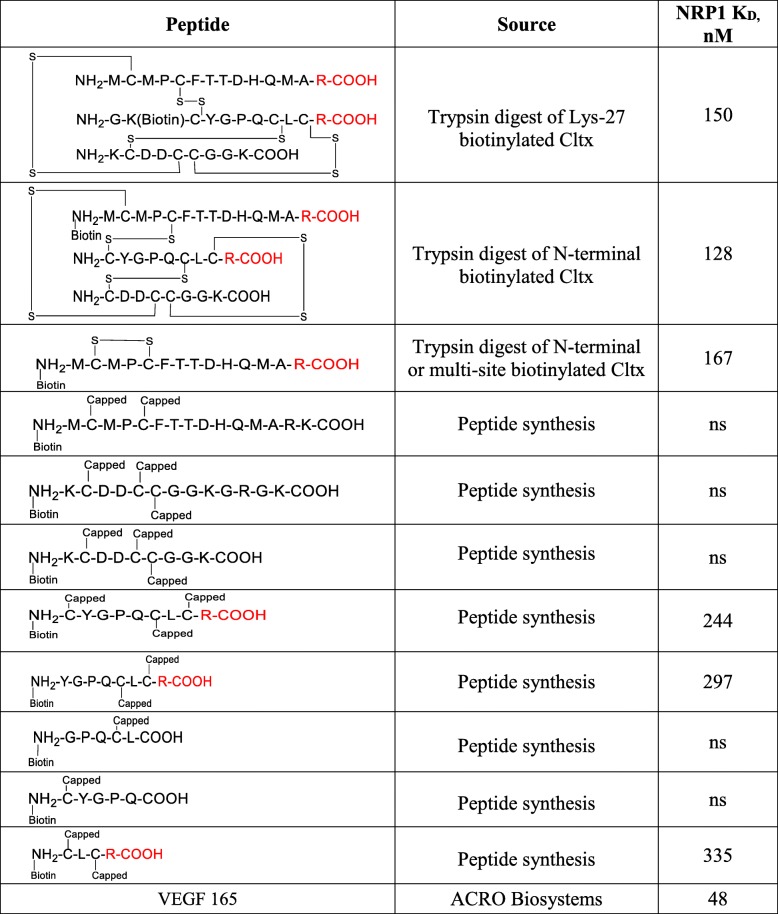
Binding of Cltx-derived peptides to NRP1. Cltx-derived peptides were generated through trypsin digest of full length native Cltx or by peptide synthesis. NRP1 binding was assessed by BLI; biotinylated peptides were attached to biosensors and evaluated for binding to NRP1 (0 to 1750 nM). Binding affinity data (K_D_) represents mean of 2 to 6 independent experiments. ns = no significant binding

### NRP1-binding peptides derived from Cltx ex vivo

Having demonstrated that NRP1 binding peptides could be generated in vitro via proteolytic digestion of Cltx, we then investigated whether Cltx-derived NRP1 binding peptides were generated in vivo. A single high dose of Cltx was administered to PC-3 tumored mice and 1 h post-treatment tumors were harvested, lysed and subjected to mass spectrometry to identify and quantify Cltx and any derived peptides. The most abundant peptide detected in tumor lysate was the administered full length Cltx which has an amidated C-terminal arginine (Fig. 3b). The next most abundant Cltx peptides terminated with deamidated C-terminal arginine residues and were 35 and 36 amino acids in length (minus initial methionine and full length respectively) (Fig. 3b). Additional shorter Cltx-derived peptides with amidated or carboxylated C-terminal arginine residues were also identified in lesser abundance (Fig. 3b).

In BLI studies, full length chlorotoxin with an amidated C-terminal arginine (Cltx-CONH_2_) did not bind to NRP1 (Fig. 3a). In contrast, full length Cltx with a carboxylated C-terminal arginine (Cltx-COOH) demonstrated dose responsive NRP1binding, with an affinity (K_D_) of ~ 240 nM (Fig. 3c). These data confirmed the absolute requirement for a ‘free’ (carboxylated) C-terminal arginine for NRP1 binding and demonstrated that such peptides were generated in tumors following administration of native Cltx (Cltx-CONH_2_). Furthermore, in competition experiments, Cltx-COOH but not Cltx-CONH_2_ competed with biotinylated VEGF for binding, suggesting that Cltx and VEGF interact with NRP1 at a similar binding site (Fig. 3d).

### Role for NRP1 in antitumor activity of ER-472

**Fig. 4:**
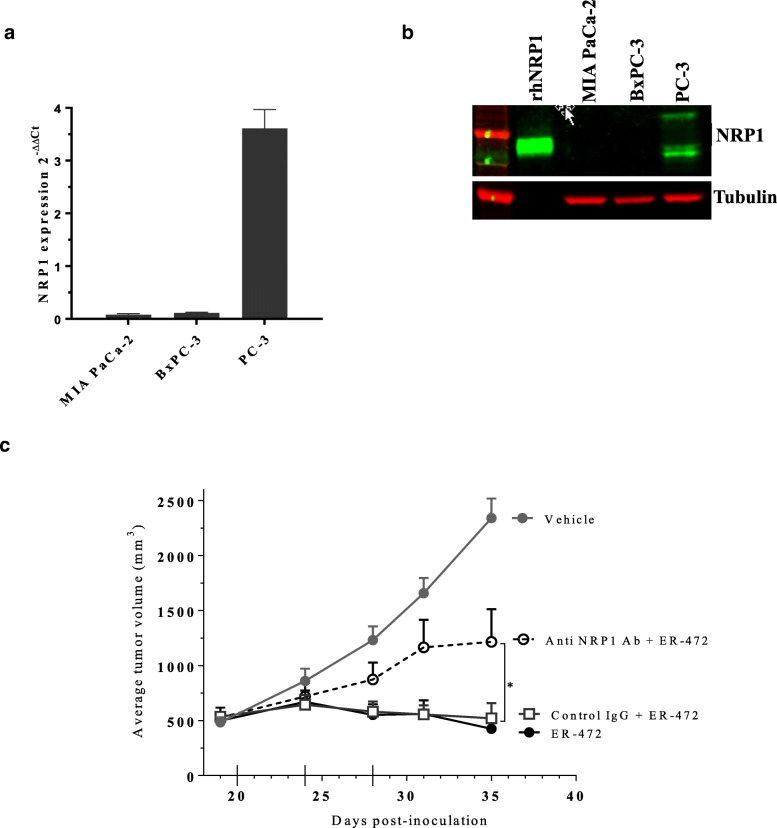
High NRP1 expression in PC-3 tumors correlates with ER-472 antitumor activity; anti-NRP1 blocking antibody treatment reduced ER-472 efficacy. **a**, expression of NRP1 mRNA in tumor lysates from 3 xenograft models. For each model *n* = 4 tumors, data represents mean ± SD. **b**, Western blot of NRP1 protein levels in tumor lysates, for each model at least 3 lysates were pooled for analysis (50 μg loaded onto gel) and data is representative of several individual experiments. Anti-NRP1 antibody detects both full length (~ 120 kDa) and soluble forms of protein (70–80 kDa); recombinant human NRP1 protein (15 ng) represents soluble/extracellular domain of NRP1. **c**, blocking anti-NRP1 antibody administered i.p. 30 min prior to ER-472 (0.9 mg/kg) diminished the PDC’s antitumor effect versus control IgG pre-treatment, *P ≤ 0.05 one-way ANOVA followed by Dunnett’s multiple comparison test

Cltx-derived peptides with free C-terminal arginine residues were identified in PC-3 tumors where they presumably bind to NRP1 following PDC metabolism. Cltx in ER-472 contributes to its antitumor activity in vivo; therefore, we sought to determine if NRP1 was also relevant for PDC therapeutic activity. First, we examined NRP1 levels in the 3 initial xenograft models and observed strong positive correlation of NRP1 expression and antitumor activity. PC-3 tumor lysates had high NRP1 mRNA and protein expression and a wide ER-472 therapeutic window, versus minimal or no NRP1 detected in the MIA PaCa-2 and BxPC-3 tumor models where ER-472 was less effective (Fig. 4a and b). To determine if NRP1 binding by Cltx-derived peptides was important for PDC antitumor activity, NRP1 was blocked using antibodies. Pre-treatment of PC-3 xenografts with anti-NRP1 blocking antibody prior to ER-472 administration resulted in a significant reduction of its antitumor effect versus control IgG or no pre-treatment (Fig. 4c). The antibody presumably blocked NRP1 binding by Cltx-derived peptides from ER-472 and diminished its activity, thus demonstrating that NRP1 contributes to ER-472 efficacy. Of note, only antibodies directed against human NRP1 attenuated ER-472 activity, while anti-mouse NRP1 antibody had a negligible effect (data not shown). This distinction implies that the NRP1 present on human PC-3 tumor cells rather than in mouse vasculature is relevant for the more potent antitumor effect of ER-472 in this model.

**Fig. 5:**
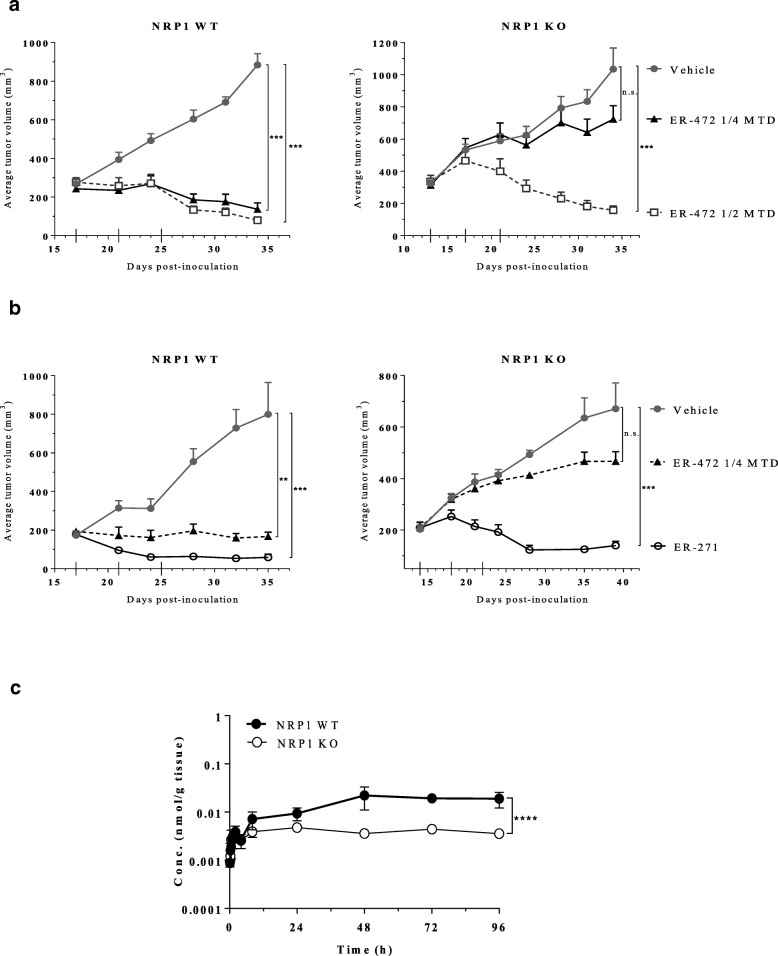
Knockout of NRP1 in PC-3 tumors blunts the antitumor effect of ER-472 through reducing active metabolite uptake into tumors. Subcutaneous tumors were established in NOD SCID mice; treatment groups were *n* = 5 or 6 and treatment schedule was Q4Dx3 (dosing days denoted with long ticks on x-axis). Tumor volume data represents the mean ± SEM. Differential sensitivity of PC-3 NRP1 WT versus KO xenografts to treatment with ER-472 (**a**) compared to ER-271 (**b**). **P ≤ 0.01, ***P ≤ 0.001, one-way ANOVA followed by Tukey’s multiple comparison test; n.s. = not significant. **c**, mean active metabolite (S-methyl cryptophycin) tumor concentration versus time profiles following a single dose of ER-472 at 0.6 mg/kg (¼ MTD). Data represents the mean ± StdDev. ****P ≤ 0.0001, unpaired t-test

To further delineate NRP1’s involvement in ER-472 activity, gene editing was used to delete NRP1 in PC-3 cells (Additional file 1: Figure S4). The antitumor effect of ER-472 was then compared in PC-3 NRP1 wild type (WT) versus knockout (KO) xenograft models. In both models ER-472 treatment at ½ MTD resulted in tumor regression (Fig. 5a). In contrast, significant divergence occurred at the ¼ MTD dose where tumor regression was observed in WT versus minimal antitumor activity in KO tumors (Fig. 5a). This result demonstrated that loss of NRP1 yields a decrease in antitumor therapeutic window for ER-472. To demonstrate that Cltx within the ER-472 conjugate was responsible for mediating the NRP1 effect, ER-472 and ER-271 (lacks Cltx peptide) were compared in the isogenic xenografts (Fig. 5b). As in the previous study, significantly higher antitumor activity was observed for ER-472 in the NRP1 WT versus KO model, while ER-271 was equipotent regardless of NRP1 status, with tumor regression observed in both models (Fig. 5b). These results affirm that NRP1 expression on tumor cells enhances the antitumor activity of ER-472 and demonstrate that Cltx within ER-472 is driving the NRP1-directed effect. Measurement of active metabolite in lysate from WT versus KO tumors following ER-472 treatment revealed a significantly higher amount (~ 4 fold more) in the NRP1 WT versus KO tumors (Fig. 5c). More metabolite in the presence of NRP1 indicates increased tumor uptake via binding of Cltx-derived peptides to NRP1 and is a compelling and plausible mechanism by which the observed amplified antitumor response was achieved.

### Correlation of ER-472 antitumor activity and NRP1 expression and relevance for cancer treatment

A strong positive correlation was noted between tumor NRP1 expression and ER-472 antitumor activity in the 3 initial xenograft models. To determine if this finding could be more broadly asserted we evaluated ER-472 activity and NRP1 expression levels in 10 additional xenograft models. Detailed results will be reported elsewhere; summarized data are provided in Table 2. Overall, a reasonable interaction was observed, with positive correlation between NRP1 expression in tumor lysate and ER-472 therapeutic window noted for 11 of the 13 total models (Table 2). To determine the clinical relevance of NRP1, expression in human tumors was assessed using TCGA RNA-seq data. A wide range of expression was observed in both tumors and normal tissue (Additional file 1: Figure S5A). In most renal cell carcinomas, sarcomas, lung adenocarcinomas and various additional tumors, NRP1 levels were significantly increased in tumor versus normal tissue, in contrast to reduced expression in certain other tumors e.g. breast cancer versus normal (Additional file 1: Figure S5A). NRP1 expression in brain tumors was further examined in light of the promising historical results for radiolabeled Cltx in this indication. In TCGA glioblastoma samples, the median NRP1 expression was significantly increased versus levels in normal brain (note: low *n* for normal brain tissue); moreover, NRP1 levels appeared to further increase upon tumor recurrence (Additional file 1: Figure S5B).

**Table 2:** Correlation of ER-472 antitumor activity to NRP1 expression in tumor lysates in multiple xenograft models. Antitumor activity was assessed for ER-472 administered i.v. on a Q4Dx3 schedule, at ¼ to full MTD doses in multiple xenograft models. NRP1 expression in tumors was assessed by mRNA analysis and/or protein detection by western blot or IHC. Reasonable correlation was determined for 11 of the 13 models

Xenograft Model	Antitumor Activity			Human NRP1 Expression	Correlation of efficacy to NRP1
	MTD	½ MTD	¼ MTD		
MIA PaCa-2	++++	–	nd	low	yes
BxPC3	++	–	nd	low	yes
PC-3	++++	++++	++	high	yes
SK-N-MC	–	nd	nd	low	yes
TC-71	–	nd	nd	low	yes
MDA-MB-231	++++	+	+	high	yes
HT-1080	+++	++	–	high	yes
U-87 MG	+++	++	+/−	high	yes
COLO 320DM	+/−	–	nd	low	yes
Hs 695 T	++++	+++	+	low^a^	no
CFPAC-1	+	–	–	low^b^	yes^b^
LOX IMVI	++	–	–	high	no
HCT 116	nd	–	nd	low	yes

^a^NRP1 expressed at high level in cell line was lost in tumors

^b^high NRP1 expression in tumor lysate by western subsequently deemed predominantly stromal by IHC; CFPAC-1 tumor cells had low NRP1 staining, a likely explanation for poor ER-472 therapeutic activity

Finally, to determine whether the NRP1 binding and tumor uptake property of Cltx could be differentiated from its reported neurological effects, we compared NRP1 binding Cltx-COOH versus native Cltx-CONH_2_ (no NRP1 binding) in a bioassay to investigate neurotoxicity. Cltx peptides were injected into crayfish which were immediately assessed for onset of paralysis. The 2 versions of Cltx were indistinguishable in this assay; both peptides induced paralysis in crayfish with a very rapid and almost identical timeframe for total incapacitation (Additional file 1: Figure S6). Thus, the neurotoxic activity of Cltx appears to be separate and distinct from the tumor targeting/uptake activity which requires NRP1 binding.

Collectively, our data demonstrate that Cltx within ER-472 acts a cryptic peptide which is metabolized to peptides with C-terminal R-COOH in the tumor microenvironment. These peptides bind to tumor cell NRP1 to increase drug uptake, which consequently boosts the antitumor effect. Based on these findings we propose that NRP1 tumor level be assessed prior to future clinical application of Cltx-based therapeutic or diagnostic agents for cancer therapy.

## Discussion

Since its discovery more than 20 years ago, Cltx has been widely used as a selective tumor targeting agent in both preclinical and human studies [1–4]. Glioblastoma, the tumor type where Cltx specificity was first identified, has been a significant target of this exploration (reviewed in 20). Extensive Cltx research is currently focused on use of the peptide as a drug/gene delivery agent in a multitude of diverse formulations including nanoparticles, liposomes, phage, etc. for glioblastoma, various additional tumor types, and other disease indications [19–21]. Since Cltx has compelling potential as a platform for specifically targeting glioma and other cancer cells, appropriate tumor stratification and patient selection are essential.

The tumor specificity of Cltx was previously attributed to the distinct targets; CLC-3, MMP2 and annexinA2, and while all 3 are likely involved in aspects of Cltx function, none fully accounts for its multiple diverse activities. ER-472, a PDC of Cltx and the potent tubulin agent cryptophycin was developed to further probe the mechanism of action of Cltx. Treatment of xenografts with ER-472 resulted in a wide range of therapeutic activity which did not correlate with tumor expression of the previously described Cltx targets. In this study, we identified the cell surface endocytic receptor NRP1 as a novel target of Cltx. A role was demonstrated for NRP1 in tumor-specific uptake of Cltx and, notably, expression of NRP1 on tumors positively correlated with the antitumor activity of ER-472. A key finding is that NRP1 binding only occurs when the C-terminal arginine of Cltx is deamidated while native Cltx from scorpion venom (Cltx-CONH_2_) does not bind NRP1. Deamidation occurs in tumor and the resultant Cltx-COOH and several derived peptides bind to NRP1. Thus, systemically, Cltx acts as a cryptic peptide, with NRP1 binding ability subsequently revealed upon reaching the tumor.

We have not examined how Cltx deamidation occurs in tumor. Deamidation is a common post-translational modification in a large percentage of proteins; it may occur as a chemical reaction or be catalyzed by deamidases (reviewed in 23). Since these enzymes have not yet been identified in eukaryotes, deamidation in the case of Cltx is likely the result of a spontaneous, non-catalyzed reaction. Studies of the anti-apoptotic protein Bcl-x_L_ have shown how deamidation can influence its function in apoptosis and autophagy and thus its oncogenic properties [22], illustrating the impact and relevance of this post-translational modification on biological activity. Importantly, when Cltx is generated recombinantly in *E. coli*, it is the deamidated, NRP1 binding, Cltx-COOH form of the peptide that is expressed. In several published reports recombinant and native Cltx peptide were used interchangeably. Based on our newly discovered NRP1 binding mechanism of action for Cltx, these earlier results, particularly those performed in vitro and with recombinant Cltx may require some re-interpretation.

NRP1 is a multifunctional protein essential for development of both CNS and vasculature [23]. It functions as a co-receptor for semaphorins and for multiple growth factors including VEGF, TGF-β, EGF, FGF, HGF and PDGF, resulting in versatile and diverse biological roles encompassing axon guidance, angiogenesis, wound healing, cancer and immunity (reviewed in [23, 24]). NRP1 is expressed by a variety of cell types; notably endothelial cells and cancer cells. Elevated levels have been reported in multiple cancer types; a wide range of expression was also observed in the TCGA analysis performed in our study, with particularly high levels noted in renal cell carcinoma and sarcoma specimens. Increased NRP1 expression is correlated with poorer prognosis and survival in many cases [25]. Pertinent to this study is the finding that NRP1 binds to peptides with an arginine rich C-terminal motif RXXR described by Ruoslahti et al. as the C-end rule [15]. Importantly, NRP1 binding results in receptor internalization, and drugs conjugated to or co-administered with the peptide are also taken into the cell by this NRP1 specific, albeit not completely delineated, active endocytosis mechanism [15, 16, 26]. This bulk transport pathway has implications in extravasation, drug delivery and penetration of tissue barriers, and because tumor cells overexpress NRP1, it may be particularly applicable for delivery of drugs into tumors, as demonstrated with ER-472. For most xenograft models, ER-472 treatment resulted in greater antitumor activity in NRP1 high versus low tumors. In addition, pre-treatment with an antibody to block tumor-expressed NRP1 reduced the potency of ER-472 in PC-3 tumors. Moreover, in isogenic PC-3 xenografts, superior antitumor activity was observed for ER-472 in NRP1 WT tumors and was demonstrated to be the direct result of increased metabolite uptake into NRP1 WT versus KO tumors, likely as a result of Cltx-NRP1 binding. Together, these findings suggest that tumors with high NRP1 expression should be selected to increase the uptake and effectiveness of Cltx-targeted agents.

In the case of Cltx and derived peptides, the previously reported RXXR motif was not required for NRP1 binding; peptides bound to NRP1 when they terminated with arginine regardless of the preceding sequence. However, the C-terminal arginine residue itself was essential since no NRP1 binding occurred when Cltx peptides terminated with any other amino acid, including lysine. This absolute requirement for arginine contrasts with reports of NRP1 binding by peptides with C-terminal lysine [15].

Binding of deamidated Cltx and derived peptides increased uptake of cryptophycin metabolite into tumors; these findings are in accord with reports that NRP1 binding increases tumor uptake of a wide variety of anticancer drugs from small molecules to larger biologicals [16]. Uptake via NRP1 interaction is not exclusive to drugs and has also been reported for virus particles, including HTLV-1 and EBV, proteins including elastase, and miRNA [17, 18, 26, 27]. Analogous to the deamidation of native Cltx in tumors, many viruses contain cryptic proteins which undergo proteolytic cleavage to become NRP1 binding and infective as a consequence, e.g. furin cleavage of EBV glycoprotein to reveal NRP1 binding and consequent virus uptake [17]. Both naked and AGO2-bound miRNAs are translocated into cells following NRP1 binding and importantly, once taken into the cell, the internalized miRNA retain their function [28]. Cltx has been extensively explored for targeted delivery of various cargo; NRP1, as described above has emerged as the portal into a significant and extensive pathway to facilitate cellular uptake. Thus, the newly discovered interaction between Cltx and NRP1 provides a well-defined mechanism to clearly explain the delivery capabilities of Cltx.

In most cases NRP1 acts with a co-receptor, transporter, etc. and often a multi-protein complex is involved in mediating cellular uptake. While identification of additional co-receptors and a detailed examination of the endocytosis mechanism upon Cltx-NRP1 binding were beyond the scope of this study, it is of interest to note that in early Cltx studies, cells treated with Cltx were reported to have reduced surface expression of both MMP2 and CLC-3 [9]. These proteins could potentially partner with or contribute in some way to the NRP1 uptake pathway which would then explain their earlier identification as Cltx targets.

During embryogenesis, NRP1 binds VEGF to mediate angiogenesis and this natural ligand (VEGF-165) bears a C-terminal arginine residue which appears essential for NRP1 binding. Loss of NRP1 results in vascular remodeling and branching defects and thus, blocking NRP1 is an attractive approach for targeting angiogenesis as an anticancer therapy. Indeed, Genentech developed an anti-NRP1 antibody which was assessed in clinical studies alone and in combination with Avastin® [29, 30]. In our studies the NRP1 binding affinity of Cltx-COOH and derived peptides was in a similar range to that observed for VEGF. Moreover, Cltx-COOH competed with VEGF for binding to NRP1, suggesting that Cltx binding occurs at the VEGF binding domain on the NRP1 receptor. Interestingly, considerable preclinical evidence in support of an antiangiogenic role for Cltx has previously been accumulated and targeting angiogenesis with unconjugated Cltx peptide was included in the original clinical strategy for TM601. In vitro, Cltx bound to HUVECs; treatment reduced MMP2 and tissue plasminogen activator secretion from these endothelial cells and also inhibited their migration and invasion [11, 12]. In vivo, angiogenesis was reduced in chorioallantoic membrane assays and matrigel plug assays; Cltx treatment also reduced neovascularization in various ocular models [12, 13]. To observe these antiangiogenic activities typically required a higher concentration of Cltx peptide than used in tumor targeting studies. NRP1 is essential for angiogenesis and Cltx possesses antiangiogenic activity; these facts, when added to our finding that Cltx competes with VEGF for NRP1 binding, lead us to the compelling conclusion that the inherent antiangiogenic properties of Cltx might also be explained by our newly discovered NRP1 binding activity.

Finally, our studies evaluating paralysis induction in crayfish demonstrate, for the first time, that the neurotoxic and tumor targeting properties of Cltx can be definitively separated. The NRP1 binding, tumor targeting capability of Cltx appears irrelevant and unnecessary for its neurotoxicity.

## Conclusions

Collectively, our studies provide an improved understanding of Cltx targeting to tumors. Binding of Cltx to the endocytic receptor NRP1 results in an increase in drug uptake, which translates to enhanced antitumor activity in vivo. The identification of NRP1 as a Cltx binding partner also contributes mechanistic insight into its antiangiogenic properties. The revised and improved knowledge of Cltx SAR strongly supports using NRP1 as a stratification and selection marker for any Cltx-containing diagnostics and therapies and should help to significantly advance research in the Cltx field.

## Additional file

Additional file 1: **Figure S1.** Structure of ER-472 PDC. **Figure S2**. Metabolism of ER-472 PDC in vivo. **Figure S3**. CLC-3, MMP2 and annexinA2 expression in MIA PaCa-2, BxPC-3 and PC-3 tumor lysates: lack of correlation with therapeutic activity. **Figure S4**. Knockout of NRP1 by gene editing in PC-3 tumor cells. **Figure S5**. NRP1 expression in human tumors, including glioblastoma. **Figure S6**. Neurotoxicity of Cltx peptides in crayfish bioactivity assay. **Table S1**. Cell growth inhibition by ER-472 in MIA PaCa-2, BxPC-3 and PC-3 cell lines in vitro. **Table S2**. Basal thiol levels in MIA PaCa-2, BxPC-3 and PC-3 tumor lysates. (DOCX 1260 kb)

## Abbreviations

BCA: Bicinchoninic acid
BLI: Biolayer interferometry
BLZ-100: Cltx covalently linked to indocyanine green (tozuleristide)
CLC-3: A member of the voltage-gated chloride channel family
Cltx: Chlorotoxin
Cltx-CONH_2_: Native Cltx from scorpion whose C-terminal arginine is amidated
Cltx-COOH: Cltx whose C-terminal arginine is carboxylated
ER-472: PDC of Cltx linked to cryptophycin
HUVEC: Human umbilical vein endothelial cell
IACUC: Institutional Animal Care and Use Committee
KO: Knockout
MALDI: Matrix Assisted Laser Desorption Ionization
MMP14: Matrix metallopeptidase 14
MMP2: Matrix metallopeptidase 2
MTD: Maximum tolerated dose
NRP1: Neuropilin-1
PDC: Peptide drug conjugate
TCGA: The Cancer Genome Atlas
TIMP: Metallopeptidase inhibitor
TM601: Refers to chemically synthesized Cltx (amidated)
WT: Wild type
