# Supplementary material for: Neuropilin-1 drives tumor-specific uptake of chlorotoxin
Source: Cell Commun Signal. 2019 Jun 17;17:67. doi: 10.1186/s12964-019-0368-9 (PMC6580635; doi:10.1186/s12964-019-0368-9)
Supplement: Supplementary file 1 — Figure S1. Structure of ER-472 PDC. Figure S2. Metabolism of ER-472 PDC in vivo. Figure S3. CLC-3, MMP2 and annexinA2 expression in MIA PaCa-2, BxPC-3 and PC-3 tumor lysates: lack of correlation with therapeutic activity. Figure S4. Knockout of NRP1 by gene editing in PC-3 tumor cells. Figure S5. NRP1 expression in human tumors, including glioblastoma. Figure S6. Neurotoxicity of Cltx peptides in crayfish bioactivity assay. Table S1. Cell growth inhibition by ER-472 in MIA PaCa-2, BxPC-3 and PC-3 cell lines in vitro. Table S2. Basal thiol levels in MIA PaCa-2, BxPC-3 and PC-3 tumor lysates. (DOCX 1260 kb) [file 12964_2019_368_MOESM1_ESM.docx]

Cryptophycin analog

Chlorotoxin

Linker

**Figure S1**

Structure of ER-472 PDC. Chemical structure of Cltx peptide linked via residue lysine 27, through a cleavable dimethyl disulfide linker, to a novel analog of cryptophycin (detailed description in [14]).

**ER-472**


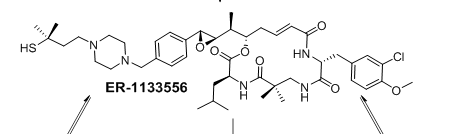

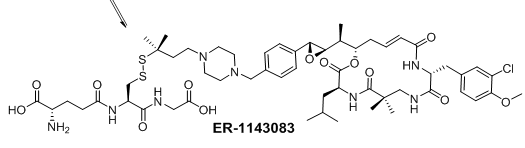


Glutathione-cryptophycin

Thiol-cryptophycin


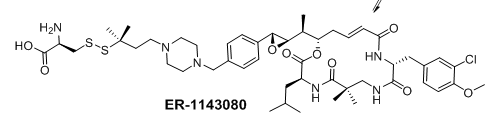


Cysteine-cryptophycin


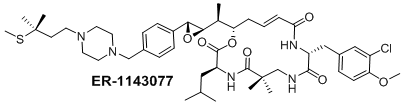


S-methyl-cryptophycin

**Methyl**

**Transferase**

**Figure S2**

Metabolism of ER-472 PDC *in vivo*. ER-472 is initially metabolized to thiol-cryptophycin by cleavage of the linker disulfide bond. Thiol-cryptophycin is unstable and quickly forms conjugates of glutathione or cysteine-cryptophycin; this step is reversible. When thiol-cryptophycin encounters methyl transferase, it is converted to S-methyl-cryptophycin in an irreversible enzymatic reaction. S-methyl cryptophycin was identified as the active metabolite of ER-472 in xenograft models.


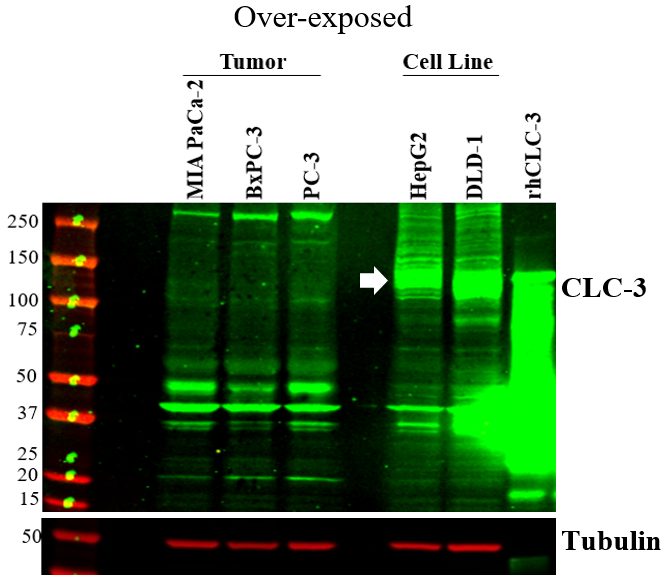
**A**


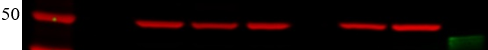


**CLC-3**

**Tubulin**


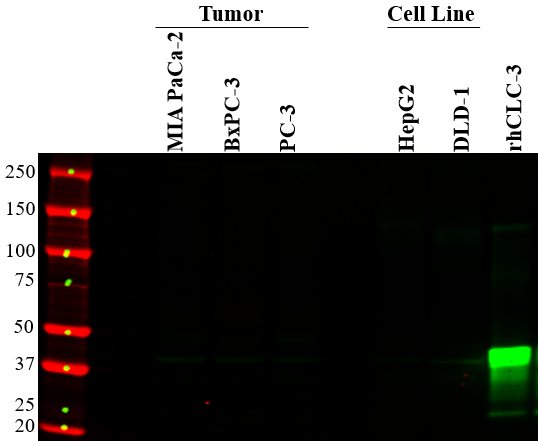


**B C**

**AnnexinA2**

**Tubulin**


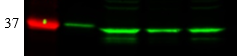


**MIA PaCa-2**

**rhAnnexinA2**

**BxPC-3**

**PC-3**


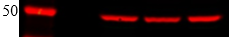

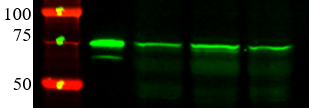


**MIA PaCa-2**

**rhMMP2**

**BxPC-3**

**PC-3**


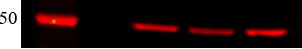


**MMP2**

**Tubulin**

**Figure S3**

CLC-3, MMP2 and annexinA2 expression in MIA PaCa-2, BxPC-3 and PC-3 tumor lysates: lack of correlation with therapeutic activity. Western blots were performed on pooled tumor lysates from the 3 models. For tumor lysates 50 µg loaded in all cases; 10 ng rhCLC-3, 3 ng rhMMP2 and 10 ng rhAnnexinA2. A, no CLC-3 protein observed in any tumor lysate; recombinant rhCLC-3 (N-terminal fragment fused to GST) was detected at correct size. A band of CLC-3 expected molecular weight (130-150 kDa) was observed in HepG2 and DLD-1 positive control cell lines upon increased exposure, but no corresponding bands in any tumor lysates which leads to conclusion that CLC-3 is minimally expressed in all 3 tumors. B, highest MMP2 expression in BxPC-3 tumor lysate, with lower and similar levels of detected in MIA PaCa-2 and PC-3 tumors. C, highest annexinA2 expression in MIA PaCa-2 with similar and lower annexinA2 protein detected in BxPC-3 and PC-3 tumor lysates.


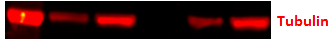

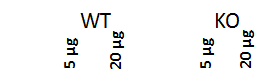

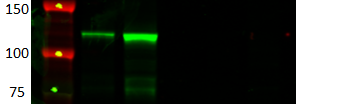


**NRP1**

**Figure S4**

Knockout of NRP1 by gene editing in PC-3 tumor cells. NRP1 knockout PC-3 cells were generated using CRISPR-mediated knockdown (cells generated and supplied by Wuxi AppTec, Shanghai, China).

**
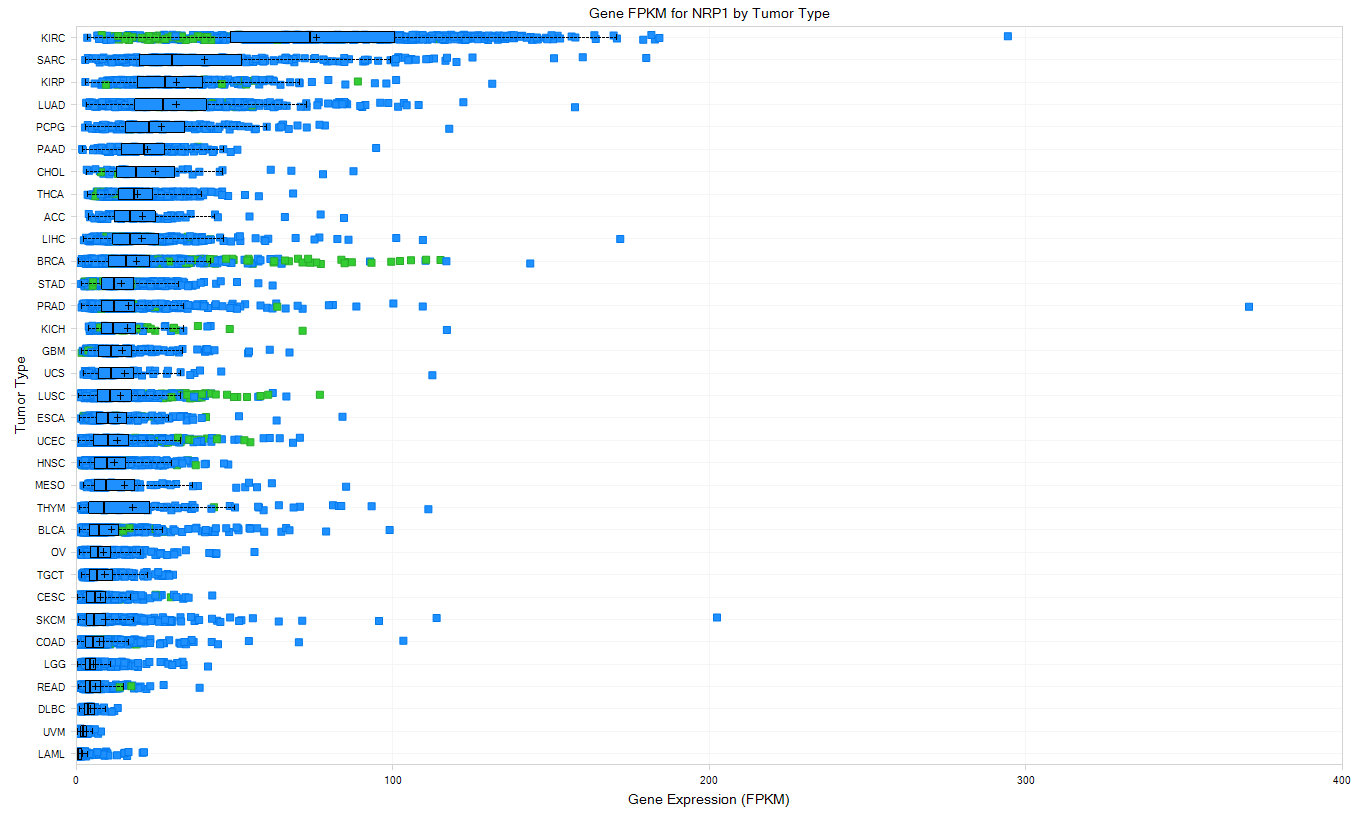
A**

**B**


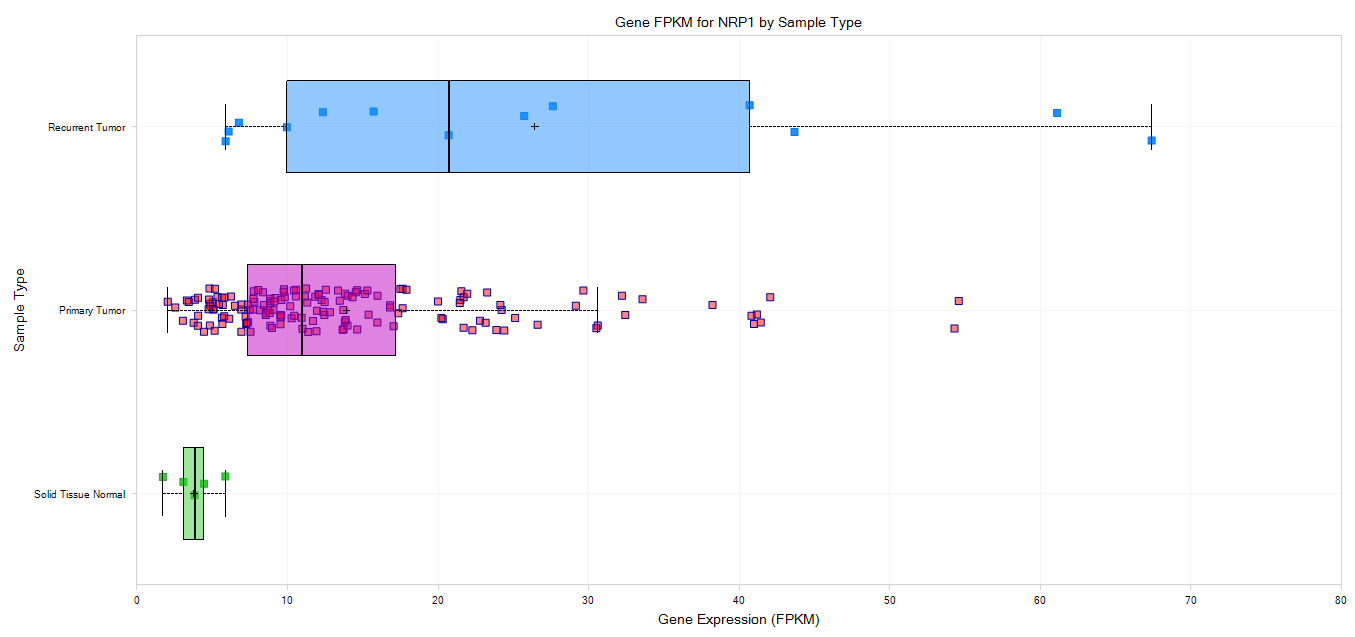


**Normal**

**Primary tumor**

**Recurrent tumor**

**0 10 20 30 40 50 60 70 80**

**Gene Expression (FPKM)**

**Figure S5**

NRP1 expression in human tumors, including glioblastoma. OncoLand’s database and visualization software (Qiagen) was used to mine TCGA RNA-Seq data and generate plots. Expression level reported as fragments per kilobase of transcript per million mapped reads (FPKM). A, NRP1 expression in all TCGA tumors reported as boxplots, line represents median expression; each blue square represents NRP1 expression in an individual tumor, while green squares reflect expression level in normal tissues. B, NRP1 expression in glioblastoma primary and recurrent tumors versus normal brain.

**Figure S6**

Neurotoxicity of Cltx peptides in crayfish bioactivity assay. Cltx with amidated (Cltx-NH_2_) versus de-amidated (Cltx-COOH) C-terminal arginine residue was injected into crayfish (20 µg per injection, n=8) and immediate response to physical challenge of continual, gentle prodding in random parts of body was recorded over time. Time from injection to induction of total paralysis was measured and the data was subjected to Kaplan-Meier survival analysis (GraphPad Prism). Median time to paralysis was almost identical; 25 versus 26 seconds for native versus de-amidated Cltx respectively.

| **Cell Line** | **Mean IC_50_**  **nM** | ***n*** |
| --- | --- | --- |
| MIA PaCa-2 | 9.7 | 3 |
| BxPC-3 | 5.2 | 2 |
| PC-3 | 8.9 | 3 |

**A**

| **Cell Line** | **48 h** | **72 h** | **96 h** |
| --- | --- | --- | --- |
| MIA PaCa-2 | 3.5 | 3.4 | 3.5 |
| BxPC-3 | 4.5 | 3.1 | 2.3 |
| PC-3 | 5.6 | 4.9 | 3.9 |

**B**

**Table S1**

Cell growth inhibition by ER-472 in MIA PaCa-2, BxPC-3 and PC-3 cell lines *in vitro*. Inhibition of cell proliferation by ER-472 over 96 h incubation was assessed in the 3 cell lines, and average IC_50_ data from 2 -3 independent assays is shown in Table 1A. B, IC_50_ values generated at multiple time points reflected no difference in the kinetics of cell growth inhibition by ER-472 between the 3 cell lines.

**A**

**B**

| **Cell Line** | **Expt. 1** | **Expt. 2** |
| --- | --- | --- |
| MIA PaCa-2 | 1.6 | 1.5 |
| BxPC-3 | 1.8 | 1.7 |
| PC-3 | 1.4 | 1.4 |

**Table S2**

Basal thiol levels in MIA PaCa-2, BxPC-3 and PC-3 tumor lysates. Two distinct assays Cayman (A) or Abcam (B) were used to measure thiol in pooled tumor lysates from all 3 models. Thiol content is reported as nmol/mg protein. Regardless of assay, tumor thiol levels were very similar in all three xenograft models. nd = not determined.

| **Cell Line** | **Expt. 1** | **Expt. 2** |
| --- | --- | --- |
| MIA PaCa-2 | nd | 1.8 |
| BxPC-3 | 2.1 | 1.9 |
| PC-3 | 1.8 | 1.9 |
